# A Case report on the Diagnosis of a Rare Pleural Tumor With Endobronchial Ultrasound: Breaking New Boundaries

**DOI:** 10.1097/MD.0000000000000561

**Published:** 2015-03-13

**Authors:** Dany Gaspard, Haroon Raja, Rohan Arya, Wissam Abouzgheib, Ziad Boujaoude

**Affiliations:** From the Division of Pulmonary and Critical Care Medicine, Cooper University Hospital, Cooper Medical School at Rowan University, Camden, New Jersey.

## Abstract

Convex endobronchial ultrasound (C-EBUS)–guided transbronchial needle aspiration (TBNA) is an effective tool for the diagnosis of hilar, mediastinal, and central parenchymal lung lesions. However, it has a limited utility for pleural-based masses. We report a unique case of a pleural synovial sarcoma recurrence that was diagnosed by C-EBUS.

The patient had a history of inguinal synovial sarcoma. He presented with cough and chest pain. Imaging of chest revealed large right pleural mass. Bronchoscopy with EBUS-TBNA diagnosed pleural recurrence of synovial sarcoma. He underwent radical resection and pathological examination confirmed the diagnosis of pleural synovial sarcoma. He experienced complete recovery and resolution of symptoms.

Synovial sarcoma should be included in the differential diagnosis of pleural masses. Convex EBUS-guided biopsies can provide adequate diagnosis of large pleural tumors adjacent to the central airways without need for more invasive diagnostic procedures.

## INTRODUCTION

Synovial sarcomas are rare tumors that typically recur in the soft tissues and the lung parenchyma, and very infrequently involve the pleural space.^1–3^ Convex endobronchial ultrasound (C-EBUS)–guided transbronchial needle aspiration (TBNA) is an effective tool for the diagnosis of hilar, mediastinal, and central parenchymal lesions, but due to its thickness, C-EBUS scope cannot usually enter the subsegmental airways and reach pleural-based masses.^4,5^ To our knowledge, there has been only 1 case of a pleural mesothelioma diagnosed by EBUS-TBNA reported in the literature.^6^ We report a unique case of a pleural synovial sarcoma recurrence that was diagnosed by C-EBUS.

## METHOD

This was a case report. Ethics committee or institutional review board approval was not obtained. It was not necessary for the case report. Informed consent was obtained from the patient.

## CASE REPORT

**FIGURE 1:**
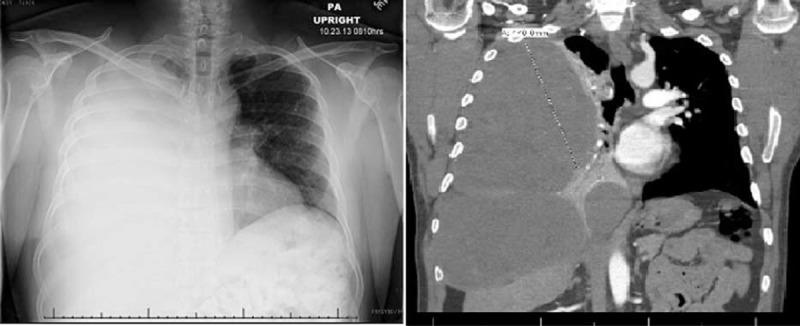
Chest x-ray and coronal view of CT scan on admission.

**FIGURE 2:**
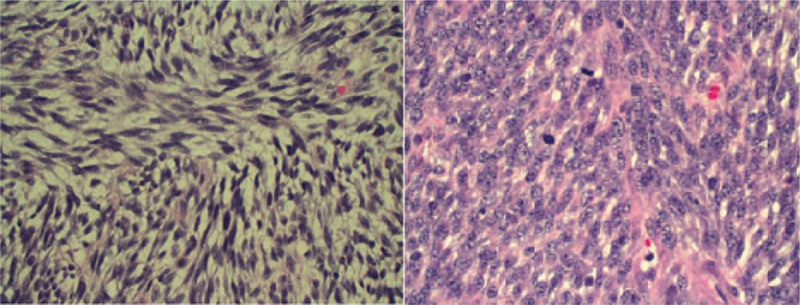
Samples of EBUS-guided biopsy of the mass (left) and surgical pathology after excision (right) both showing similar fragments of tissue composed of cellular fascicles of uniform spindle cells with darkly staining nuclei and very scant cytoplasm.

**FIGURE 3:**
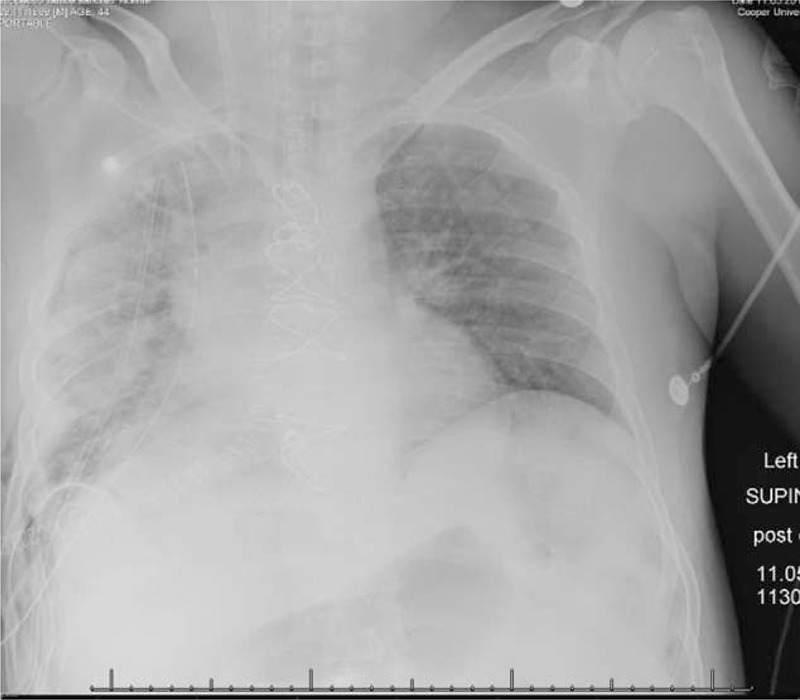
Postoperative chest x-ray with reexpansion of a large portion of the right lung.

A 50-year-old man with history of inguinal synovial sarcoma resected 3 years prior presented for right-sided chest pain and a persistent cough of 3 months duration. Chest x-ray showed an almost complete whiteout of the right lung. CT scan of the chest showed a very large, multilobed, pleural-based tumor collapsing most of the right lung as well as small mediastinal and hilar lymphadenopathy (Fig. 1). He underwent flexible bronchoscopy with C-EBUS. There was no endobronchial involvement. No hilar or mediastinal lymph nodes were visualized except for a 10-mm right paratracheal node, which was sampled. When the bronchoscope was advanced into the right lower lobe, a large heterogeneous mass was visualized with EBUS, and multiple TBNA samples were obtained. No complications were encountered. Cytopathology analysis showed spindle cells positive for vimentin and caldesmon and negative for EMA, pancytokeratin, HMB45, S100, desmin, SMA, CD34, and CD117, all findings consistent with synovial sarcoma (Fig. 2). He was evaluated by oncology and cardiothoracic surgery. Two weeks later, he underwent debulking surgery with resection of most of the mass. The tumor was completely of pleural origin without parenchymal involvement. Postoperative x-rays showed reexpansion of the lung (Fig. 3), and after a few days of recovery he noted improvement in his cough and shortness of breath. Surgical pathology confirmed the initial diagnosis. He was started on chemotherapy.

## DISCUSSION

Fibrosarcomas are soft tissue tumors of fibroblastic origin. Once considered to be the most frequent type of sarcomas, they have since then been found to be exceedingly rare and constitute only about 1% of all soft tissue sarcomas.^1^ Approximately, 11,000 cases are diagnosed in the United States every year. There are over 100 different histologic subtypes; synovial sarcomas are one of the more common ones. The most frequent sites of initial involvement include lower extremities, head and neck, trunk, upper extremities, mediastinum, and abdomen. These tumors have a high risk of metastases and a high frequency of recurrence.

Several large series have been published looking at sites of recurrence. The largest one comes from the National Cancer institute in 1985.^2^ The most frequent site of metastasis was by far the lung parenchyma, followed by local recurrence, then bones and lymph nodes. Other smaller series reported similar findings.^3^ The pleural space has not been reported as a site of metastasis of these tumors. This is the first unique feature of this case. Secondly, and perhaps more interestingly, an adequate diagnosis was made by endobronchial ultrasound. EBUS-TBNA has emerged in the past few years as an effective tool to diagnose and stage a large number of chest malignancies. It allows sampling of mediastinal, hilar, and proximal endobronchial masses,^4,5^ but its utility for reaching pleural lesions is limited. This is mainly due to the thickness of the scope which usually limits its ability to enter the subsegmental airways. There have been multiple reports of diagnosis and staging of pleural malignancies with EBUS; however, this was possible only indirectly, through transbronchial aspiration of enlarged mediastinal lymph nodes and not the pleural mass itself.^6,7^ Only 1 case of endobronchial sampling and diagnosis of a pleural-based mesothelioma is reported in the literature.^8^ Although typically transthoracic biopsy would have been the first step in evaluation of our patient's tumor, we elected to start with flexible bronchoscopy for 2 reasons: First, the transthoracic biopsy of pleural sarcomas is known historically to be often difficult with a low yield. Second, and most importantly, we wanted to assess for endobronchial involvement given the size and near total collapse of the lung and evaluate ipsilateral and contralateral lymph nodes for staging purposes. Fortunately in our case the mass was large enough to be at proximity to the airways and was visualized by EBUS. The samples obtained allowed an adequate diagnosis of the histology of the mass and helped guide the next steps of management without the need for more invasive procedures.

In a recent review article from Great Britain, José et al concluded that since the advent of EBUS, fewer patients needed to undergo mediastinoscopies for diagnosis and staging of lung cancer.^9^ Other authors have discussed that it complements and may one day replace the use of blind transbronchial biopsies.^10^ Our case describes yet another clinical application of EBUS and adds further to the rapidly growing body of the literature supporting its utility in diagnosing both malignant and nonmalignant diseases of the chest.^11^ Traversing the visceral pleura transbronchially appears to be safe but certainly careful postprocedural monitoring of these patients is warranted because of the added risk of iatrogenic complications.

## CONCLUSION

Synovial sarcoma should be included in the differential diagnosis of pleural masses. Convex EBUS-guided biopsies can provide adequate diagnosis of large pleural tumors abutting proximal airways without need for more invasive diagnostic procedures.

## References

[1] Bahrami A Folpe AL Adult-type Fibrosarcoma: a reevaluation of 163 putative cases diagnosed at a single institution over a 48-year period. *Am J Surg Pathol* 2010; 34:1504–1513. 20829680 10.1097/PAS.0b013e3181ef70b6

[2] Vezeridis MP Moore R Karakousis CP Metastatic patterns in soft-tissue sarcomas. *Arch Surg* 1983; 118:915–918. 6307217 10.1001/archsurg.1983.01390080023007

[3] Potter DA Glenn J Patterns of recurrence in patients with high-grade soft-tissue sarcomas. *J Clin Oncol* 1985; 3:353–366. 3973646 10.1200/JCO.1985.3.3.353

[4] Tournoya KG Rintoulb RC van Meerbeecka JP EBUS-TBNA for the diagnosis of central parenchymal lung lesions not visible at routine bronchoscopy. *Lung Cancer* 2009; 63:45–49. 18514365 10.1016/j.lungcan.2008.04.004

[5] Boujaoude Z Dahdel M Pratter M Endobronchial ultrasound with transbronchial needle aspiration in the diagnosis of bilateral hilar and mediastinal lymphadenopathy. *J Bronchology Interv Pulmonol* 2012; 19:19–23. 23207258 10.1097/LBR.0b013e3182442b89

[6] Hamamoto J Notsute D Diagnostic usefulness of endobronchial ultrasound-guided transbronchial needle aspiration in a case with malignant pleural mesothelioma. *Intern Med* 2010; 49:423–426. 20190476 10.2169/internalmedicine.49.2825

[7] Rice DC Steliga MA Endoscopic ultrasound-guided fine needle aspiration for staging of malignant pleural mesothelioma. *Ann Thorac Surg* 2009; 88:862–868. 19699913 10.1016/j.athoracsur.2009.05.022

[8] Lococo F Rossi G Agostini L “Dry” pleural mesothelioma successfully diagnosed on endobronchial ultrasound (EBUS)-guided transbronchial needle aspiration (TBNA). *Intern Med* 2014; 53:467–469. 24583437 10.2169/internalmedicine.53.1563

[9] José RJ Shaw P Impact of EBUS-TBNA on modalities for tissue acquisition in patients with lung cancer. *QJM* 2014; 107:201–206. 24259720 10.1093/qjmed/hct233 PMC3930811

[10] Xia Y Wang KP Transbronchial needle aspiration: where are we now? *J Thorac Dis* 2013; 5:678–682. 24255782 10.3978/j.issn.2072-1439.2013.09.11 PMC3815731

[11] Dincer HE Linear EBUS in staging non-small cell lung cancer and diagnosing benign diseases. *J Bronchology Interv Pulmonol* 2013; 20:66–76. 23328148 10.1097/LBR.0b013e31827d1514

